# Gargoyles: An Open Source Graph-Based Molecular Optimization Method Based on Deep Reinforcement Learning

**DOI:** 10.1021/acsomega.3c05430

**Published:** 2023-09-28

**Authors:** Daiki Erikawa, Nobuaki Yasuo, Takamasa Suzuki, Shogo Nakamura, Masakazu Sekijima

**Affiliations:** † Department of Computer Science, Tokyo Institute of Technology, 4259-J3-23, Nagatsuta-cho, Midori-ku, Yokohama 226-8501, Japan; ‡ Academy for Convergence of Materials and Informatics (TAC-MI), Tokyo Institute of Technology, S6-23, Ookayama, Meguro-ku, Tokyo 152-8550, Japan; § Department of Life Science and Technology, Tokyo Institute of Technology, 4259-J3-23, Nagatsuta-cho, Midori-ku, Yokohama 226-8501, Japan

## Abstract

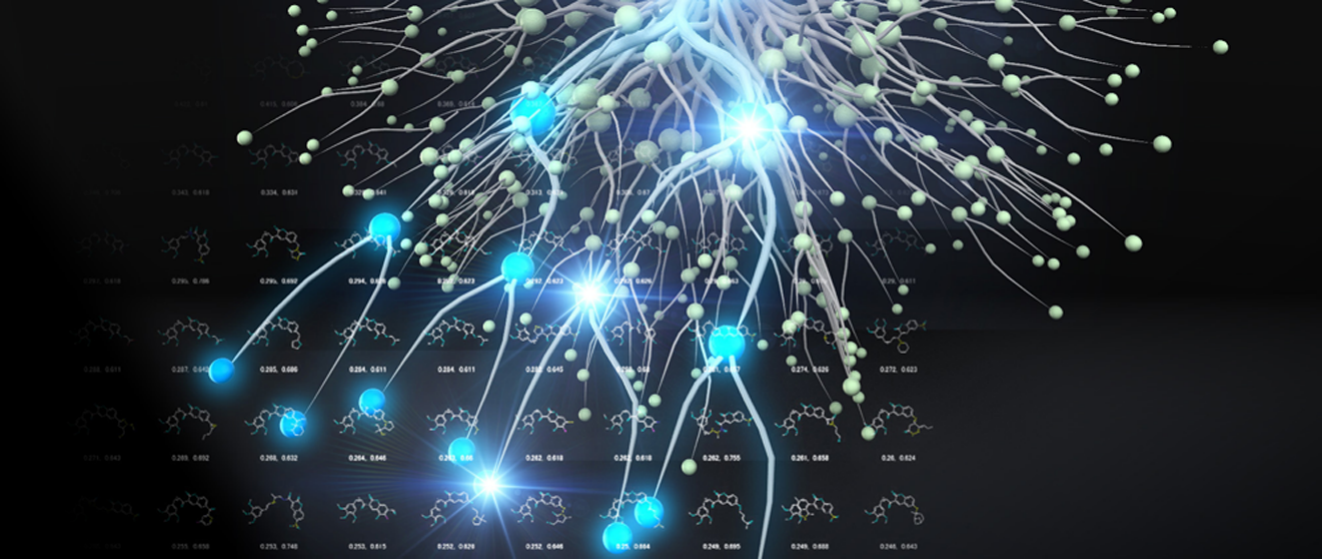

Automatic optimization methods for compounds in the vast compound space are important for drug discovery and material design. Several machine learning-based molecular generative models for drug discovery have been proposed, but most of these methods generate compounds from scratch and are not suitable for exploring and optimizing user-defined compounds. In this study, we developed a compound optimization method based on molecular graphs using deep reinforcement learning. This method searches for compounds on a fragment-by-fragment basis and at high density by generating fragments to be added atom by atom. Experimental results confirmed that the quantum electrodynamics (QED), the optimization target set in this study, was enhanced by searching around the starting compound. As a use case, we successfully enhanced the activity of a compound by targeting dopamine receptor D2 (DRD2). This means that the generated compounds are not structurally dissimilar from the starting compounds, as well as increasing their activity, indicating that this method is suitable for optimizing molecules from a given compound. The source code is available at https://github.com/sekijima-lab/GARGOYLES.

## Introduction

Drug discovery is the process of identifying potential new therapeutic compounds, peptides, or antibodies for the treatment of diseases. It involves a series of steps beginning with the identification of biological targets, followed by the development of hit compounds, optimization of lead compounds, and finally, preclinical and clinical testing of drug candidates.^1^

Hit compounds are compounds that show initial activity against target proteins involved in diseases, such as 3CL protease,^2^ spermidine synthase,^3,4^ dephospho-CoA kinase,^5^ and nicotinamide adenine dinucleotide (NAD)-dependent deacetylase Sirtuin 1,^6^ and have potential to be developed as drugs. Hit compounds are found through various screening methods, including high-throughput screening, which searches for targets from a large compound library.

Hit-to-lead is the process of optimizing a hit compound into a lead compound, which is a more promising and optimized drug candidate. In this phase, the properties of the hit compound are optimized to improve its efficacy and safety as a drug candidate. The lead compound then undergoes further testing and development to determine its suitability as a drug candidate.^7^

Typically, 200,000 to 1 million compounds are screened first. Then, more than 100 compounds are screened in hit-to-lead and lead optimization to narrow the molecules down to one or two candidates.^1^ Subsequently, it has been shown that approximately 1 in 10 (10.4%, *n* = 5820) of the leads and all indications that entered Phase 1 were approved by the FDA.^8^ Thus, the project often fails and has a low success rate.^9,10^ If even a portion of these processes could be assisted by in silico methods, it would save a tremendous amount of time and expense.^11^ With recent developments in computers and algorithms, the application of computer science technology to drug discovery has been explored, and the efficiency and quality of the drug discovery processes have been improved.^12−17^

Molecular generative models are computer-based methods related to hit compound discovery and lead optimization.^18^ Molecular generative models have the advantage of being able to efficiently explore a huge chemical space and generate novel compounds with desirable properties using machine learning. They also have the advantage of avoiding explicitly dealing with complex chemical knowledge by using large compound data sets to train machine learning models. Several methods have been proposed to optimize molecules according to evaluation functions.^19^ The variational autoencoder can be used to generate molecules by modeling in the latent space.^20−24^ Then, optimization can be performed with the gradient method, leveraging the fact that the latent variables are continuous with the model that predicts the evaluation value from the latent variables. Such approximate models of the evaluation function are generally used in molecular optimization. String representation of molecules^25^ (SMILES) can be generated by long short-term memories (LSTMs) of recurrent neural networks^26−29^ (RNNs). In this case, optimization is performed by retraining the generative model using the data set in which molecules without the target property are removed from the generated molecules by the approximate model of the evaluation function. Optimization using generative adversarial networks (GANs) is performed in the same manner.^30^ Some optimization methods are also based on reinforcement learning. One popular approach is to represent molecular generation as a Markov decision process in which the state is a molecule, and the action is the addition of atoms (or fragments) to approximate the policy function with a machine learning model.^31,32^ This approach has the advantage of directly optimizing the evaluation function compared to an approximate model. In addition to the policy gradient method, there are other optimization methods such as Q-learning and Monte Carlo Tree Search (MCTS).^33−35^ While this is suitable for generating compound libraries used to search for hit compounds, it is not suitable for cases such as lead optimization, where a candidate compound has already been narrowed, and optimization is performed on that compound. Several methods focusing on starting generation from arbitrary molecules have been proposed.^29,36^ Mol-CycleGAN is one of the methods that start generation from a given molecule. The method is trained using sets of molecules before and after optimization based on the CycleGAN generation scheme.^37^ Optimization is performed via latent variables, but according to experimental results, it is inferior in terms of performance compared to methods using reinforcement learning. MERMAID is the most relevant method for this study, and it starts generation from an arbitrary molecule by editing SMILES with MCTS and LSTM.^38^ However, the generated molecules often deviate significantly from the initial molecules as the optimization progresses. In this study, we developed a molecular optimization method based on molecular graphs, starting from an arbitrary molecule to be explored by MCTS. The use of molecular graphs allows for high similarity to the starting compound, while MCTS allows for efficient generation without prior learning of a specific evaluation function. In addition, a graph neural network model trained on the compound data set is used to enhance the efficiency of the search. The search is conducted per fragment, but the fragments to be added are generated atom by atom. This allows more appropriate fragments to be added to the current molecule while avoiding the lack of diversity caused by using a fixed fragment vocabulary.

**Table 1:** Properties of the Fragment Data Set Used for Training the GCN Model

metrics	
molecular weight	158 ± 45
number of atoms	10.6 ± 3.2

**Table 2:** Properties of Starting Molecules Used in Unconstrained Optimization Experiments

metrics	
QED	0.653 ± 0.028
molecular weight	345 ± 69
number of atoms	24.1 ± 5.0

**Table 3:** Properties of Starting Molecules Used in Constrained Optimization Experiments

metrics	
*P* log *P*	–12.26 ± 5.74
molecular weight	317 ± 71
number of atoms	22.6 ± 5.2

## Results

### Unconstrained Optimization

**Figure 1:**
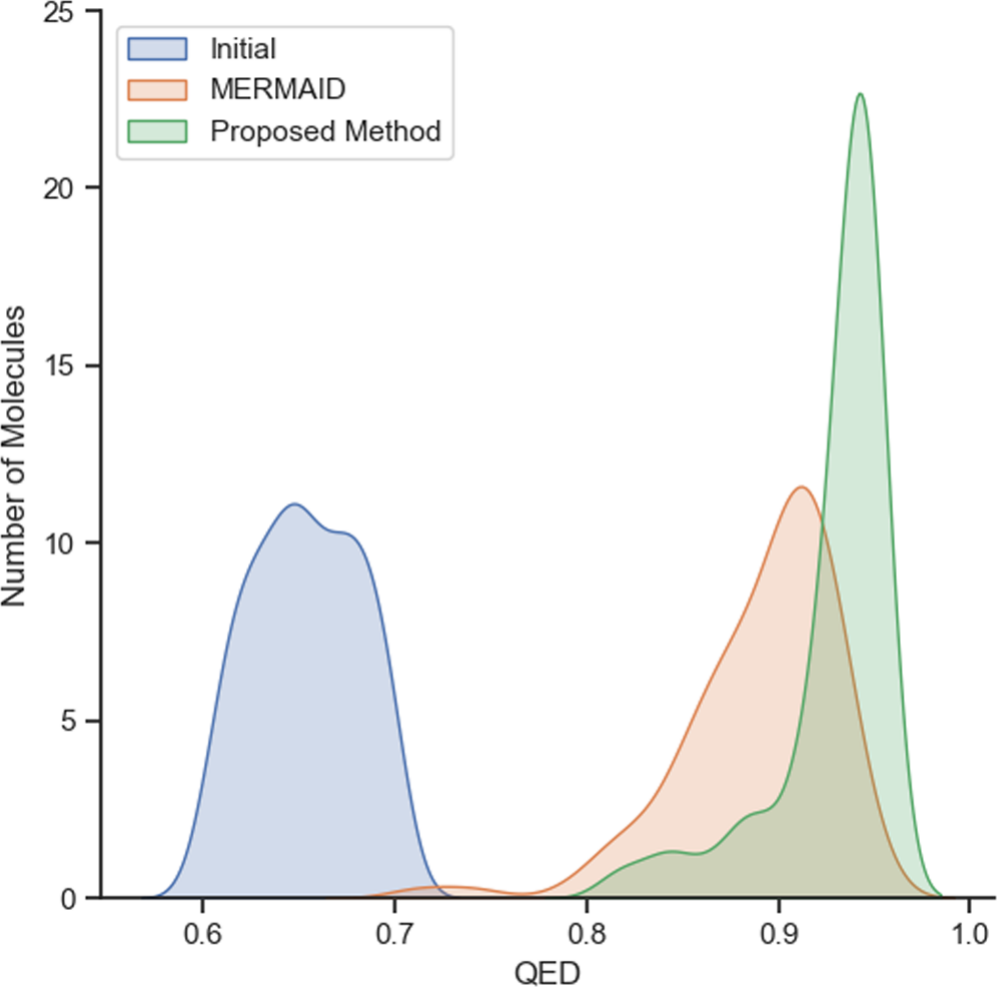
Distribution of QED for molecules optimized by the proposed method and MERMAID. The distribution of the starting molecules is represented as ‘initial.’

Table 4 shows that the quantum electrodynamics (QED) value was more than 0.92 for the top few cases, which is approximately sufficient for optimization. Furthermore, MERMAID,^38^ which is the baseline for comparison in this study, had a lower evaluation function value of 0.3 to 1.4. This is also visualized in Figure 1. The difference between the two methods is the representation of molecules: MERMAID uses SMILES, and the proposed method uses molecular graphs. Considering the ZINC data set as an example of ”data size,” the average number of nodes in a molecular graph was approximately 24, while the average number of tokens in SMILES was approximately 40. Since both methods search only one node/token per step, this method was considered to search more when compared with the same number of steps. Additionally, while SMILES generated invalid SMILES, the molecular graph was always valid, which is another reason why this method was able to search more. Note that SMILES with RNNs generally runs faster than molecular graphs with graph convolutional networks (GCN) when compared in a single step.

**Table 4:** QED Values of Unconstrained Optimization

method	first	second	third	50th	avg top 50
seed	0.653 ± 0.028	−	−	−	−
MERMAID	0.890 ± 0.041	0.875 ± 0.045	0.865 ± 0.047	0.750 ± 0.048	0.794 ± 0.047
proposed method	0.928 ± 0.032	0.925 ± 0.033	0.923 ± 0.036	0.891 ± 0.051	0.905 ± 0.045

**Figure 2:**
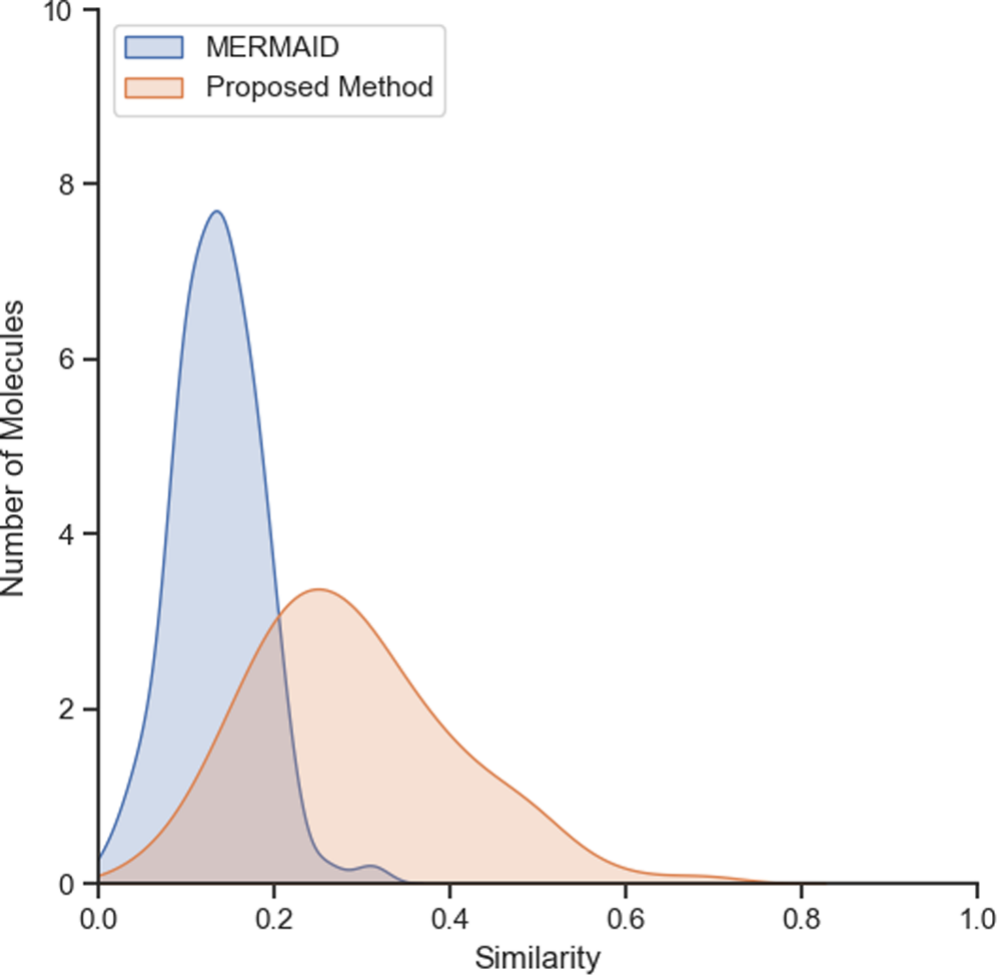
Similarity distributions from the starting molecule to the molecules optimized with the proposed method and MERMAID.

In terms of validity, this method employing molecular graphs always produced valid molecules (i.e., validity = 1), while MERMAID using SMILES produced strings that do not satisfy the SMILES grammar (i.e., validity = 0.7). Novelty and uniqueness were all 1 or nearly 1, confirming that there were no problems when viewed from these perspectives. The similarity shows higher values for this method clearly (Figure 2). Given that the similarity for all pairs of molecules in the ZINC data set of approximately 250,000 molecules was 0.144, the molecules generated by this method can be considered to be similar to the starting molecules. The SA score was clearly better for this method. This may be due to the fact that the molecular graphs used in this method allow only the addition and deletion of single bonds, making it difficult to create complex structures.

**Figure 3:**
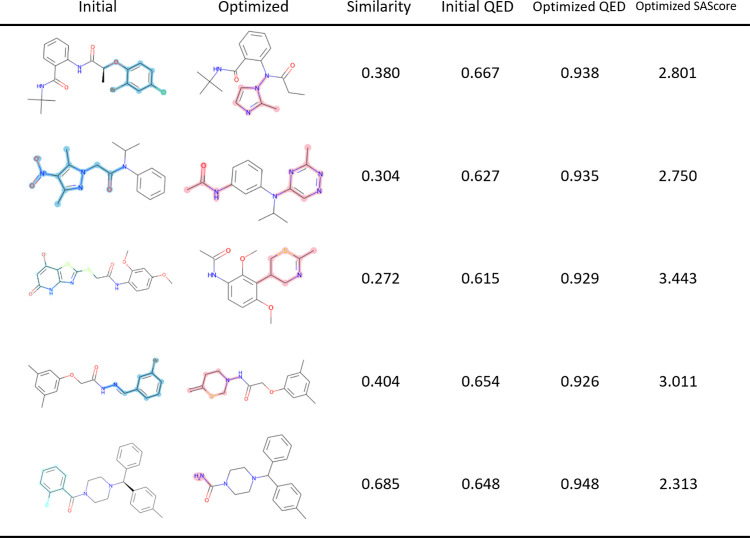
Molecular pairs before (left) and after (right) optimization. The blue areas represent substructures that were removed from the initial structure, while the red areas represent substructures that were added during the optimization process.

**Figure 4:**
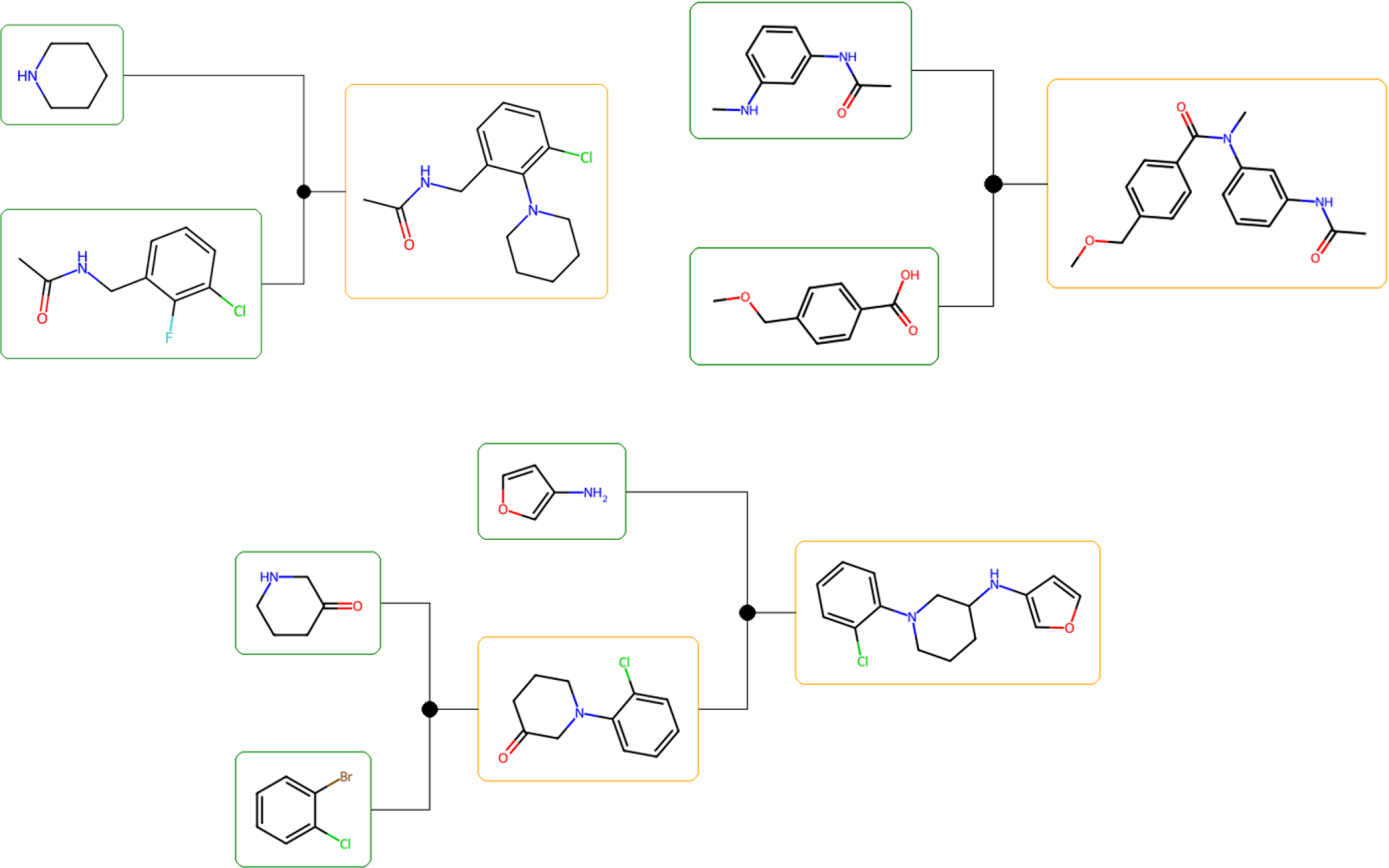
Examples of synthetic pathways produced by AiZynthFinder for optimized molecules. Compounds in the green frame are registered as commercial reagents in AiZynthFinder.

Examples of molecules generated by this method are shown in Figure 3. It can be seen that the addition and deletion of a few fragments from the starting molecule resulted in a molecule with improved QED values while maintaining a high degree of similarity. One of the reasons for the higher similarity obtained with this method compared with MERMAID using SMILES is that the addition and deletion of fragments are limited to single bonds, thereby avoiding the direct editing of the ring structure as in MERMAID. However, this restriction of actions has a disadvantage in terms of diversity because complex rings cannot be generated (in the fragment-wise search). Examples of molecules classified by Graph as having a synthetic pathway are shown in Figure 4.

### Constrained Optimization

Table 5 shows that the improved value of *P* log *P* was slightly less than that of GraphAF but was still sufficiently better than that of the other three methods. The success rate was approximately 1, indicating that *P* log *P* can be optimized regardless of starting molecules. Note that the comparison of the methods here is not completely fair because they have different generation schemes. For example, GraphAF and GCPN generate molecules after fully optimizing the policy model through reinforcement learning, whereas MERMAID and the proposed method exhibit a disadvantage in that they do not train the network for a specific evaluation function. Furthermore, the constraint of similarity is a favorable task for MERMAID and the proposed method in terms of the generation scheme.

**Table 5:** Results of Constrained Optimization Experiments^a^

methods	improvement	similarity	success (%)
GCPN	0.79 ± 0.63	0.68 ± 0.08	100
mol-cycle-GAN	1.22 ± 1.48	0.69 ± 0.07	19.3
graphAF	4.98 ± 6.49	0.66 ± 0.05	96.88
MERMAID	1.99 ± 1.74	0.62 ± 0.02	85.3
proposed method	4.18 ± 5.84	0.62 ± 0.06	99.3

a Improvement represents the increase from the original *P* log *P*, and success represents the ratio of successfully optimized molecules where improvement is a positive value. In addition to the mean, standard deviations are also shown for improvement and similarity.

### Activity Optimization

**Figure 5:**
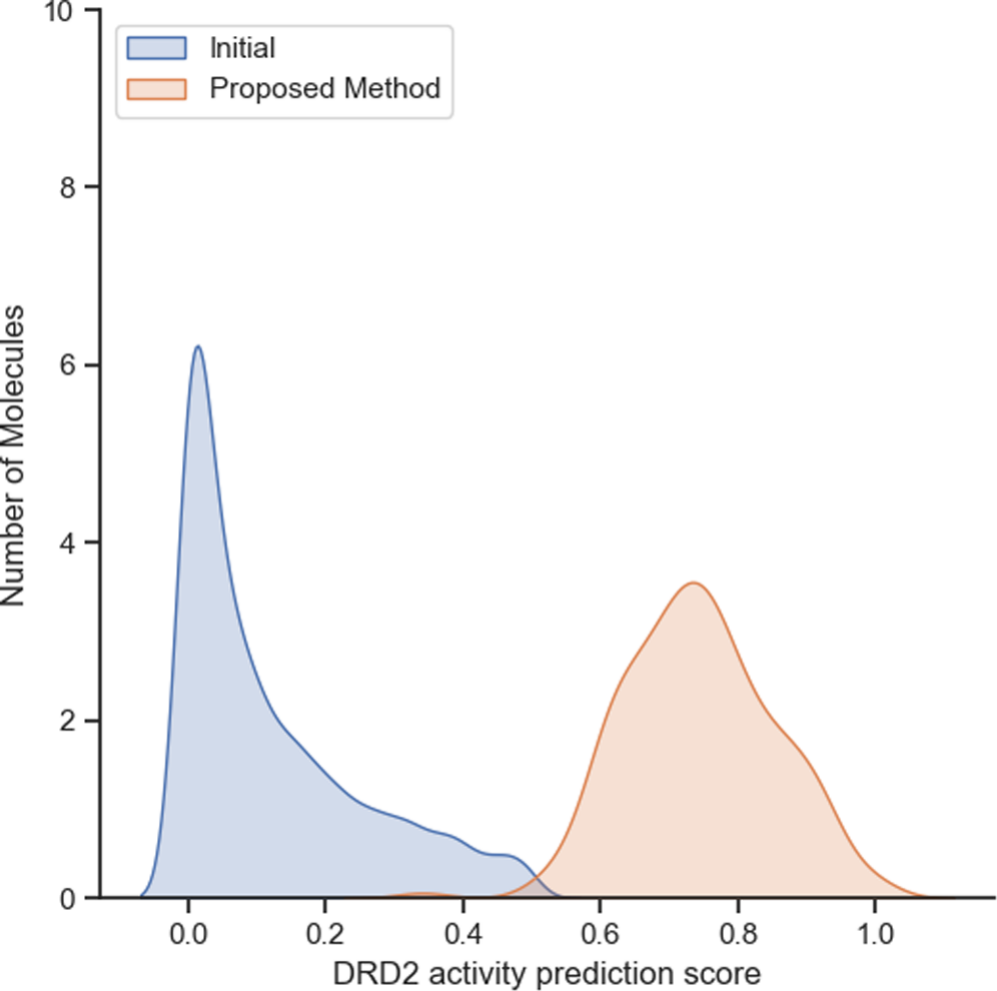
Distribution of predicted activity score for DRD2 for molecules optimized by the proposed method. The distribution of the starting molecules is represented as ‘initial.’

Table 7 shows that seed molecules with an average low activity prediction score of 0.122 were optimized down to molecules with an activity prediction score of 0.782, which is close to molecules with *K_i_* < 100 nM. The percentage of molecules with an activity prediction score of 0.5 or higher among the generated molecules is also shown. The results of other methods are also shown but should only be used as a reference since the seed molecules are different. The optimization can also be seen in Figure 5, which shows the distribution of activity prediction scores for seed and optimized molecules. The execution time for optimization depends on both the number of steps and the evaluation function. For example, this compound optimization experiment in dopamine receptor D2 (DRD2) took an average of 175 s for each iteration of MCTS. The number of atoms of the generated fragments was 6.43 ± 2.65, and the molecular weight was 97 ± 39. Thus, it was found that a wide variety of fragments can be generated. This is because fragment generation is performed in a Monte Carlo tree search; therefore, different fragments are always generated at different steps, and the number of fragments generated does not differ significantly. It should be noted that these results include errors in the activity prediction model.

## Discussion

We compared and evaluated the initial molecule and the generated molecule in terms of the similarity given by the Tanimoto coefficient based on the ECFP4 fingerprints, except for the value of the evaluation function. However, in practice, lead optimization is expected to maintain not only the similarity but also the potency and selectivity to the target. Although the similarity may be correlated to these properties, it is more appropriate to evaluate in terms of potency and selectivity. Considering the comparison of the initial molecule and the generated molecule by such a metric for drug efficacy, this method is not expected to retain a high percentage of the original drug efficacy. The search space of this method is narrowed down to a molecule-like graph by the GCN model and biased by MCTS toward regions with higher values of the evaluation function. Thus, if specific properties such as drug effects are to be considered, then they must be explicitly handled in the evaluation function. Masking the evaluation function to preserve important substructures with respect to the property of interest is also effective.

## Conclusions

Most molecular generative models do not consider starting from a given molecule and are not appropriate for situations such as lead optimization. Additionally, existing methods that start generation from a given molecule have problems such as low similarity to the starting molecule. Therefore, in this study, we developed a molecular optimization method that starts with generation from arbitrary molecules based on molecular graphs. Optimization is performed in a fragment-wise tree search according to a given evaluation function. Fragments are generated individually by MCTS in an atom-by-atom manner. The GCN model trained on the fragment data set is used to improve the efficiency of fragment generation. In an experiment of optimizing QED as an example of the evaluation function, the generated compound not only improved the value of the evaluation function sufficiently but also had a high similarity to that of the starting compound. In addition, it was confirmed that this method searches near the starting compound compared with existing methods. For the synthetic pathway prediction, the results also show the advantages of a graph-based approach. Experiments demonstrated that this method allows for optimization not only in QED but also in aspects such as activity toward the target protein. Thus, this method is considered to be suitable for processes such as lead optimization, where compound candidates have already been obtained. Furthermore, this method can be used to mask important structures identified beforehand, such that they remain unchanged. This method can be applied to other applications, such as providing more promising compounds to compound libraries for virtual screening.

## Methods

The molecular generation method developed in this study is a molecular-graph-based optimization method for a given arbitrary molecule based on an evaluation function. The method performs a tree search with the molecule to be optimized as the root by adding and deleting fragments based on the upper confidence bound (UCB) score. Fragments to be added are generated atom by atom with MCTS, and the efficiency is enhanced by using a GCN model. Generating fragments atom by atom allows the molecule to be further optimized locally (with only partial conformational changes).

### Monte Carlo Tree Search

MCTS^39^ is a model-based reinforcement learning algorithm and has been used as an effective policy improvement operator in deep reinforcement learning methods such as AlphaGo.^40^ MCTS searches for the optimal sequence of state and action sequences by sequentially constructing a search tree with the initial state as the root. Each node has a state value and a number of visits, each initialized with 0. The following four steps are repeated in one cycle until a given convergence condition is satisfied.

1. Selection: select one leaf node from the current search tree according to a criterion known as Tree Policy.
2. Expansion: add a child node to the selected node.
3. Simulation: the newly added node is expanded to the terminal state (not added to the search tree) according to the Default Policy, which is called rollout.
4. Backpropagation: update the evaluation value of the node corresponding to the path from the root node to the selected node using the obtained evaluation value and increase the number of visits plus one.

Tree Policy must take into account exploration and exploitation and the UCB1 score^41^ expressed in the following manner:
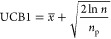
where *x̅* is the average reward for the self-node *n*, and *n*_p_ is the number of visits to the self-node and the parent node. Default Policy is guaranteed to converge to the optimal solution with a sufficient number of steps even with random selection, but this is not feasible in practice. Machine learning models have often been used in recent years to search efficiently.

### GCN

GCN^42^ is a neural network comprising layers that perform convolution operations defined over graph data. Convolution cannot be simply applied to graphs, unlike images or series, in which the relationship between neighboring elements is not fixed. There are two types of convolutions on graphs: one dealing with signals over graphs and the other based on the spatial structure of graphs. Here, we describe graph convolution defined on the space used in this study. The output of the l layer of node i depends only on its neighbors and is defined as follows:^43^
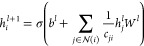


where *h*^*l*/*l*+1^ is the output of the *l*/*l* + 1 layer, *b*^*l*^ is the bias of the *l* layer, *W*^*l*^ is the weight parameter,  is the neighbor of the node *i*, *c*_*ji*_ is the product of the order roots of the nodes *j* and *i*, and σ is the activation function.

### Fragment-Wise Search

**Figure 6:**
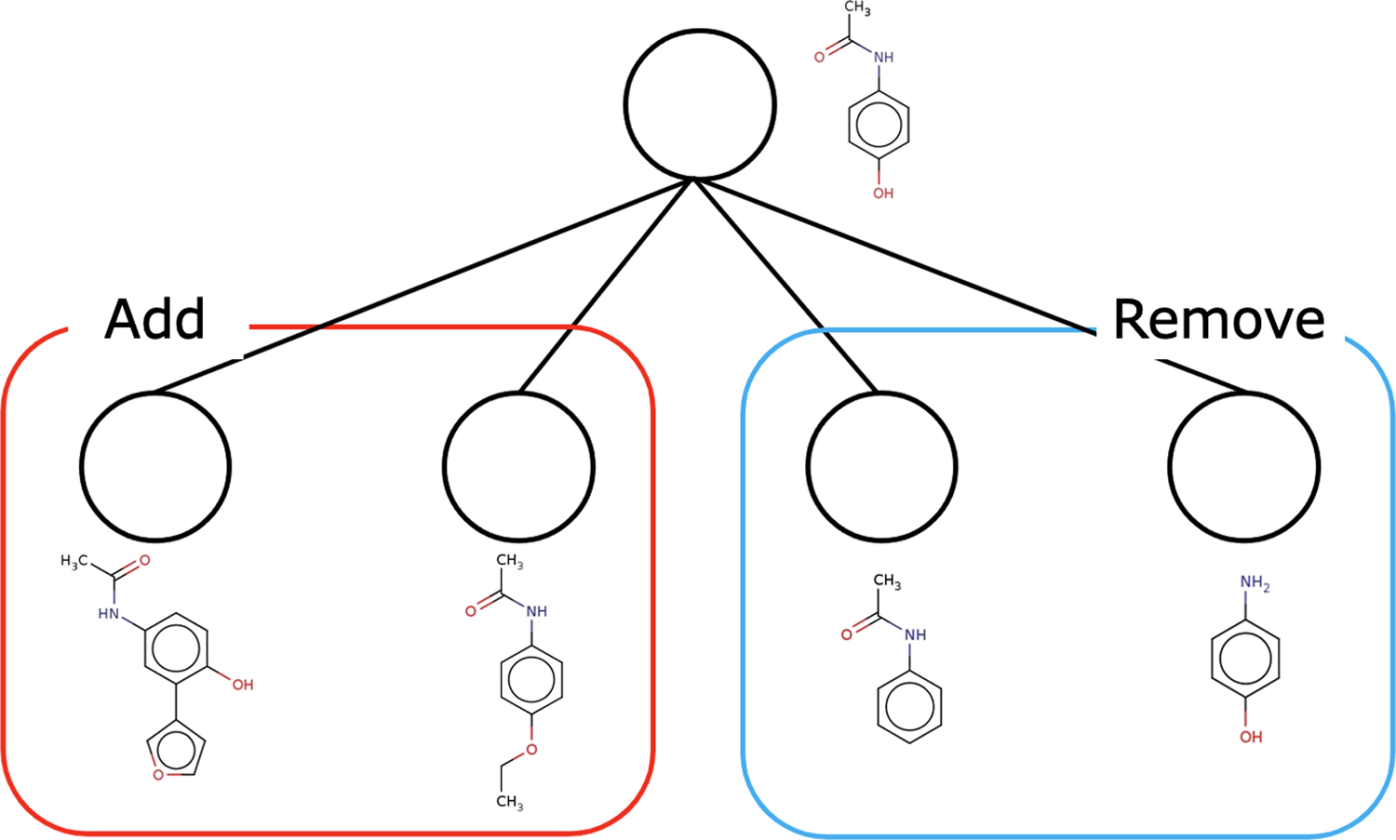
Optimization is performed by tree search with addition and removal of fragments.

The fragment-wise search (Figure 6) is the core process of the method. Optimization is performed by editing the molecular graph fragment by fragment, given an arbitrary molecule and an evaluation function as input. The state of each node in the search tree corresponds to a molecule with a state value and a number of visits. The action in the tree search is to remove and add fragments. The tree search with the starting molecule as the root searches with the following process as one cycle. The molecule corresponding to the newly added node is treated as the optimized molecule.

**Figure 7:**
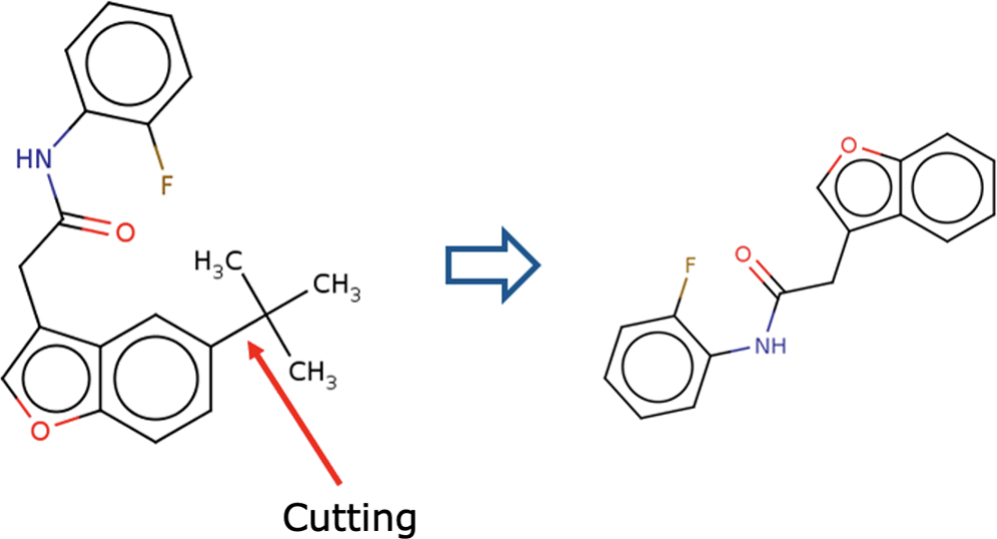
Removal of fragments in a fragment-wise tree search.

**Figure 8:**
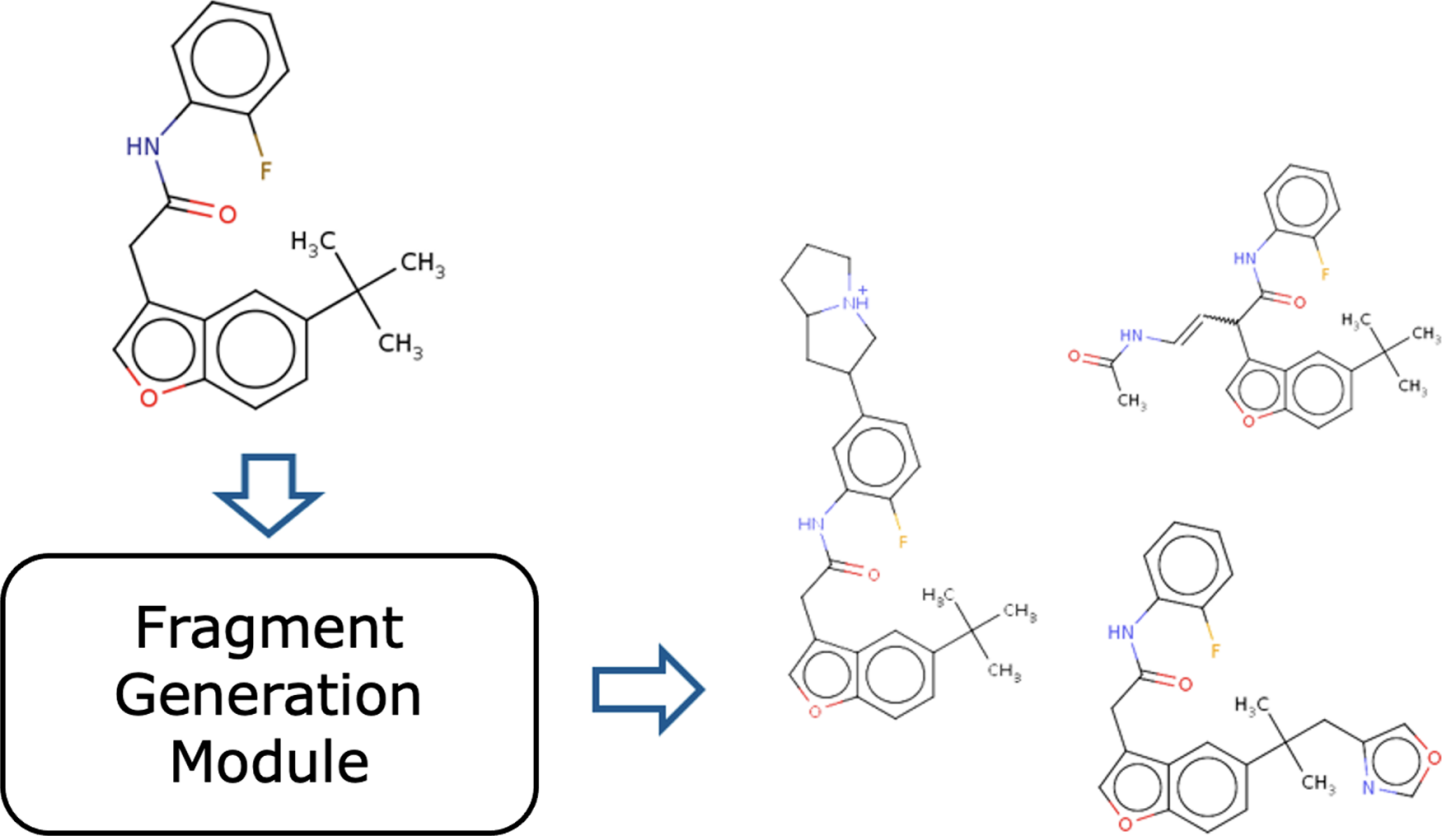
Addition of fragments in a fragment-wise tree search.

1. Selection: one leaf node is selected from the current search tree based on the UCB1 score.
2. Expansion: the molecules obtained by adding and removing fragments from the molecules corresponding to the selected nodes are added as child nodes.
  - Removing fragments (Figure 7): for all single bonds in the molecule corresponding to the selected node, the node with the higher number of atoms is added as a new child node with the fragment-removed molecule only if it splits into two when breaking the bond.
  - Adding fragments (Figure 8): the molecule adding the fragment is generated by the fragment generation module and is treated as a molecule corresponding to the child node added to the search tree. The details are described in the atom-wise search.
  The number of child nodes for removal is finite and small, e.g., an average of approximately 9 in the ZINC data set,^44^ but the number of child nodes for addition is significantly large due to the number of molecules generated by the fragment generation module. Therefore, the selection of some molecules based on some criteria is necessary. In this study, random selection and a criterion for a high value of the evaluation function, such as ϵ-greedy,^45^ were selected.
3. Evaluation: the evaluation value for the molecules of the newly added child node is used as the reward. The reason for this is that unlike board games, where the terminal state can be easily determined, it is difficult to perform a rollout in molecular generation due to the ambiguity of the terminal state. For example, the benzene ring is nonterminal when naphthalene is generated, but it is terminal when the benzene ring is generated. Existing methods use machine learning models to determine if the state is terminal or to fix the number of steps. However, when the search starts from an arbitrary molecule and proceeds in the direction of increasing and decreasing atoms, as in this method, determining whether the search is terminated is difficult because the initial molecule is already completed. Another reason is that deep nodes are likely to deviate significantly from the initial molecule, which is not in accordance with the purpose of the study.
4. Backpropagation: for all nodes in the path from the root node to the selected node, the state value and number of visits are updated using the maximum value of the rewards calculated in the simulation step. The following transformations are applied to keep the reward value in the range of −1 to 1:
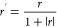

### Atom-Wise Search

**Figure 9:**
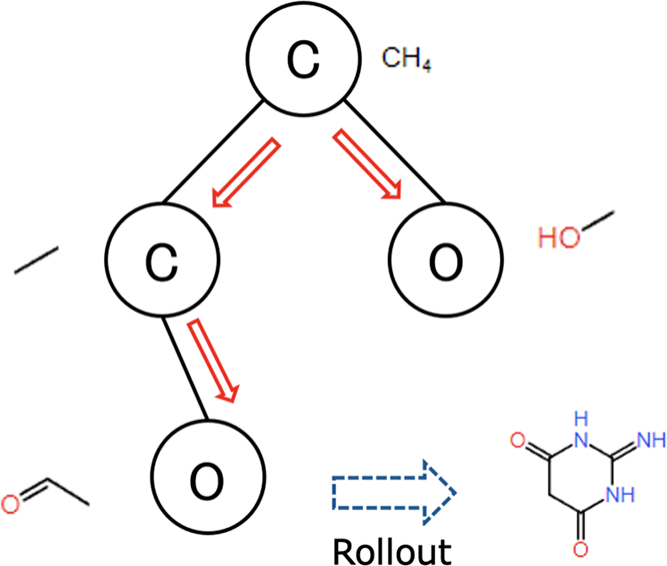
Fragment generation in an atom-wise manner.

Atom-wise search corresponds to the generation of newly fragmented molecules in the expansion step of MCTS in the fragment-wise search (Figure 9). One path corresponds to one fragment by assigning one atom to one node. In one step, a new atom and the bonds associated with that atom are predicted to be added to the fragment in the intermediate state. Atoms that cannot be further bonded are excluded, considering the valence rule in the expansion step. Atom-wise MCTS is executed as one cycle of the following process.

1. Selection: as with fragment-wise MCTS, one leaf node is selected from the current search tree based on the UCB1 score.
2. Expansion: the molecular graph corresponding to the selected node is used as input to predict the next atom and bond with the GCN model. New molecules added the predicted atoms and bonds are added to the search tree as child nodes of the selected node.
3. Simulation: rollout is performed using the same GCN model used in the expansion step to evaluate the added child nodes. Unlike the fragment-wise search, the fragments are generated from scratch; therefore, the terminal state can be defined in the same way as for existing methods. A state is treated as a terminal if the GCN model predicts an empty atom or when no bonds are predicted. The generated fragments are added to the molecule selected in the selection step of MCTS in the fragment-wise search to obtain a new molecule, and the value of the evaluation function for that molecule is used as the reward.
4. Backpropagation: similar to the fragment-wise search, the value and number of visits are updated for all nodes in the path from the root node to the selected node.

### Fragment Generation by the GNN Model

**Figure 10:**
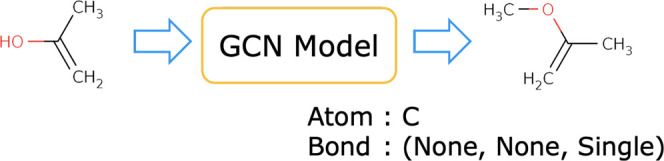
Flow of the GCN model.

This section details the GCN model used for the expansion and rollout of nodes in MCTS in the atom-wise search. An overview of the process is shown in Figure 10.

### Flow of Prediction by the GCN Model

The GCN model comprises three modules that perform feature extraction, atom prediction, and bond prediction.

1. Feature extraction: the feature extraction module takes a molecular graph x as input and outputs hidden states of graph and nodes *h*_g_ and *h*_n_ through a GCN. The graph hidden state is computed from the node hidden states using the aggregation function as follows:
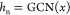

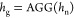
2. Atom prediction: the atom prediction module takes the hidden state of the graph as input and outputs the type of atom as a probability by passing it through the fully connected layer (FC_a_(·)). The dimension of the output is (atom type) +1, which is the sum of the number of atom types and the label indicating the termination.
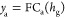
3. Bond prediction: the bond prediction module takes the predicted atom and hidden states of the nodes as input and predicts the bonds through the RNN layer. The initial state vector *s* of the RNN is the concatenation of the vector obtained by transforming the predicted atoms with the embedding layer (Emb(·)) and the hidden representation of the graph *h*_g_. The input of the RNN is the hidden state vector of nodes *h*_n_ arranged as a series of data according to the BFS order of nodes in the input graph. The output is the type of bonds as a probability, whose dimension is (number of bond types) +1 including a label, indicating that there is no bond.
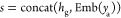

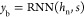

The node features of the molecular graph are one-hot vectors of atom types. Aromatic bonds are not explicitly represented by representing aromatic rings as a Kekule structure in which three single and double bonds appear alternately.

### Training

The GCN model used for fragment generation needs to be trained on the fragment data set and not the whole molecule. Fragment data sets are created from existing molecule data sets by the following procedure.

**Figure 11:**
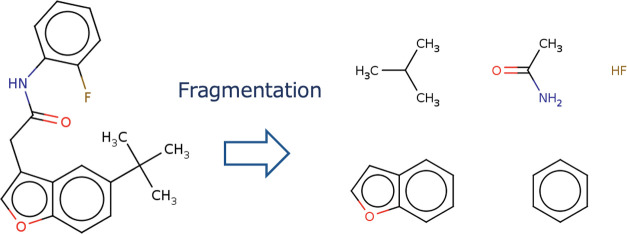
Examples of fragments used to train GCN model. In this example, cutting all of the bonds between rings and nonrings yields five fragments.

1. For each molecule, a set of fragments is obtained by breaking all bonds that connect rings and nonrings (Figure 11).

The other processes are the same as those for general training of models.

## Experimental Section

### Training of the GCN Model

This section describes the architecture of the GCN model used in the atom-wise search and its training. The GCN model is used in fragment generation and aims to capture the features of molecules without maximizing the efficiency of a specific evaluation function. Therefore, the same model was used for all experiments in this study.

#### Architecture of the GCN Model

**Figure 12:**
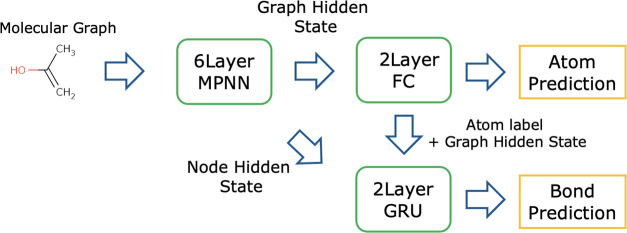
Architecture of the GCN prediction model.

As described in the Methods section, the model comprises three modules: feature extraction, atom prediction, and coupling prediction, and the details of each are described below. The architecture of this model is shown in Figure 12.

- Feature extraction: the input molecular graph has nine-dimensional node features and three-dimensional edge features. The transformation with 6-layer MPNN outputs a 128-dimensional node hidden state vector *h*_n_ (*N*_n_, 128), where *N*_n_ is the number of nodes in the input graph. Sum pooling is then applied to obtain the graph hidden state vector *h*_g_ (1, 128).
- Atom prediction: the atom prediction module comprises two fully connected layers. The hidden layer has 64 dimensions and uses ReLu functions as activation functions. A softmax function is applied to this output *y*_a_ to obtain the probability of each atom. The output is ten-dimensional, comprising an empty atom, meaning termination and nine types of atoms.
- Bond prediction: the bond prediction module comprises a two-layer GRU^46^ followed by a two-layer fully connected layer. The initial state vector of the GRU is a concatenation of the graph hidden state vector *h*_g_ and the 64-dimensional embedded representation of the predicted atoms Emb(*y*_a_) and is converted to 256 dimensions in a single fully connected layer. The output of the GRU is transformed to *y*_b_ by the fully connected layer, with the hidden layer having 64 dimensions and ReLu function as the activation function. The dimension of outputs is four, including the label, indicating no bond. The probability of a bond is obtained by applying a softmax function.

#### Training Data

The ZINC database^44^ used for training is a database for virtual screening and contains over 750 million molecules. Approximately 250,000 molecules, which are commonly used in molecular generative models, were used for training. This data set is the same as that used in ChemTS^34^ and is publicly available at https://github.com/tsudalab/ChemTS. 22,234 fragments obtained by applying the procedure described in the Methods section to each molecule were used for training. Properties of the fragment dataset used for training the GCN model are shown in Table 1. An example of the fragments used for training is shown in Figure 11.

#### Training Process

The labels are atom type and bond type, and each loss was calculated as a cross-entropy loss function, where the sum of losses is the overall loss. The ratio of train–test data was 4:1, and parameters were updated with the Adam optimizer. The model was trained in a teacher-forcing^47^ manner, in which label data were used as inputs when there was a dependency between the inputs. The input for the bond prediction was not the output of the atom prediction module but the label of the atom because the prediction of the bond depends on the prediction of the atom. The other training settings were as follows.

- Learning rate: 0.0001
- Batch size: 128
- Epoch: 50

In the experiment, the parameters for 20 epochs of training were used.

### Unconstrained Optimization

In this experiment, an optimization based on a single evaluation function was performed. The evaluation function was QED.^48^ The reason QED was used here is not that it is sufficient to increase QED in lead optimization but that it is easy to calculate, and QED is commonly used in the evaluation of molecular generative models. In addition, the actual pathway by which the optimized molecule can be synthesized is an important point to be considered and cannot be easily obtained as in the case of QED. Several methods have been proposed to predict synthetic pathways using machine learning.^49,50^ We used AIZynthFinder to determine whether the optimized molecule has a practical synthetic pathway.

#### Settings

One hundred molecules with QED in the range of 0.6 to 0.7 were randomly selected from the ZINC data set and used as starting molecules. The properties of the starting molecules used in this experiment are shown in Table 2. For each molecule, a 20-step fragment-wise search was performed. Then, the atom-wise search was performed in 100 steps for each molecule. As a result, the optimization was performed in 2,000 steps.

#### Evaluation Metrics

There are several evaluation metrics and benchmarks for molecular generative models, and the appropriate metrics vary depending on the method and purpose. The following evaluation metrics were selected based on the purpose of this study.

- QED: evaluate the degree to which QED has increased compared to the original molecule. Among the group of molecules generated for each starting molecule, the top 1, 2, 3, and 50th values and the average value for the top 50 molecules are used.
- Validity: it is the ratio of valid molecules that satisfy the valence rule among the generated molecular graphs. In this case, it is always 100% because molecular graphs are used.
- Novelty: it is the ratio of molecules not present in the training data set among the valid generated molecules. It is low for models that copy molecules in the training data set directly.
- Uniqueness: it is the ratio of nonoverlapping molecules among the valid generated molecules.
- Similarity: calculate the similarity to the starting molecule. Given the purpose of this study, a higher value is desirable. Similarity is calculated as the Tanimoto coefficient based on the ECFP4 fingerprint.

### Constrained Optimization

In this experiment, we compared the proposed method with existing methods through the commonly used task of optimizing PLogP under similarity constraints. Eight hundred molecules with the lowest PLogP from the ZINC data set were optimized as starting compounds. GCPN, Mol-CycleGAN, MERMAID, and GraphAF were used as comparison methods.

#### Settings

Eight hundred molecules with the lowest *P* log *P* selected from the ZINC data set were used as the starting molecules. The properties of the starting molecules used in this experiment are shown in Table 3. For each molecule, a 2-step fragment-wise search was performed. Then, the atom-wise search was performed in 100 steps for each molecule. As a result, optimization was performed in 200 steps.

#### Evaluation Metrics

Evaluation was based on the difference in *P* log *P* between the starting molecule and the generated molecule (improvement), the similarity to the starting molecule (similarity), and the ratio of examples in which *P* log *P* increased while satisfying the similarity constraint (success rate).

### Activity Optimization

In this experiment, activity optimization toward dopamine receptor D2 (DRD2) was performed. The activity of DRD2 is obtained by an activity prediction model using Random Forest. The comparison method is Mol-CycleGAN as a method that starts with a specific molecule, as a graph-based generation method.

#### Settings

ChEMBL data set is used as training of activity prediction model and seed of DRD2 activity optimization. Activity for DRD2 is considered active when *K_i_* < 100 nM and inactive otherwise. The ratio of train/test split is 8:2. The output of the activity prediction model with 0.88 ROC AUC test score is a real number between 0 and 1, and a higher number indicates higher activity.

200 molecules randomly selected from the subset of ChEMBL data set (*K_i_* > = 100 nM and predicted activity score <0.5) are used as seed molecules, and optimization is performed with a 2-step fragment-wise search and a 100-step atom-wise search. Table 6 shows an overview of the DRD2 data set.

**Table 6:** Properties of the DRD2 Data Set

metrics	
number of active (*K_i_* < 100 nM)	3981
number of inactive	7839
predicted activity score (*K_i_* < 100 nM)	0.885 ± 0.203

#### Evaluation Metrics

Evaluation was based on the predicted activity score of optimized molecules and similarity between seed and optimized molecules (Table 7).

**Table 7:** Results of DRD2 Activity Optimization Experiments^a^

metrics	proposed method	Mol-CycleGAN
predicted activity score	0.782 (seed: 0.122)	0.362 (seed: 0.179)

a The predicted activity score is calculated by the activity prediction model. The results of Mol-CycleGAN are obtained from each original paper. Note that the seed compounds of Mol-CycleGAN were different.
